# Tube feeding associated postoperative intussusceptions: A single center case series study

**DOI:** 10.1097/MD.0000000000017783

**Published:** 2019-11-01

**Authors:** Liangshuo Hu, Guozhi Yin, Dong Zhang, Zhimin Geng, Jigang Bai

**Affiliations:** Department of Hepatobiliary Surgery and Institute of Advanced Surgical Technology and Engineering, The First Affiliated Hospital of Xi’an Jiaotong University, Xi’an, China.

**Keywords:** complication, gastrointestinal anastomosis, gastrojejunostomy tube, pancreatoduodenectomy, postoperative intussusceptions

## Abstract

**Rationale:**

Postoperative intussusception in adults is a rare but serious complication after gastrointestinal anastomosis surgery. Postoperative intussusception in adults caused by tube feeding was rarely been reported before. The aim of the current study was to summarize the clinical data on a group of patients with tube feeding associated postoperative intussusceptions. The possible etiology and preventive measures will also be discussed.

**Patient concerns:**

During the period from May 2013 to January 2018, patients who received gastrointestinal anastomosis in our center were retrospectively reviewed. Preoperative variables including standard demographic and pathological characteristics as well as the treatment and prognosis were also analyzed.

**Diagnoses:**

Tube feeding associated postoperative intussusceptions.

**Interventions:**

7 patients were identified with tube feeding associated postoperative intussusceptions with a prevalence of 0.38%. Intussusceptions occurred from 10 to 69 days (median 25.7 days) postoperatively in an acute form.

**Outcomes:**

None of the patients had spontaneous reduction and all patients underwent surgery. Antegrade efferent limb intussusceptions were found in all the cases. Intussusception occurred at efferent loop at 23.6 cm (range 15–60) from the gastrointestinal or Braun anastomosis. None of the patients was found recurrence throughout the follow-up period.

**Lessons:**

In contrast with other postoperative intussusceptions, the tube feeding associated postoperative intussusceptions have special clinical manifestations. It is more likely to occur in early period of time after the surgery and in an acute form. Surgical correction is recommended for most of patients. Several measures have been proposed to prevent such complications after gastrointestinal surgery, however more research and information are still needed.

## 1 Introduction

Intussusception is known as the telescoping (inversion) of a bowel segment of the gastrointestinal tract into an adjacent segment. While in children, intussusception is the most common cause of bowel obstruction, it constitutes only 1% to 5% of adult intestinal obstructions.^[1,2]^ Although being very rare, postoperative intussusception in adults is a serious complication after gastrointestinal anastomosis surgery including Billroth II or Roux-en-Y reconstruction. It represents an uncommon cause of post-operation intestinal obstruction with a reported prevalence of 0.07% to 1.2%.^[3,4]^ Adult bowel intussusceptions usually have a trigger factor and are mostly associated with a bowel lesion.^[5,6]^ However, the etiology of postoperative intussusceptions in adults is still not entirely clear.

Tube feeding is one of the enteral feeding strategies after pancreatoduodenectomy (PD) or other surgical procedures in gastrointestinal reconstruction. Although controversial, it is advocated that the routine use of tube feeding could help with blood sugar control, reduction of infection rates, and shortened length of hospital stay.^[7–9]^ Gastrojejunostomy (GJ) tube associated small bowel intussusceptions have been reported in pediatric patients.^[10]^ Hughes reported 40 cases of intussusceptions which were associated with GJ tubes and the morbidity is 16%.^[10]^ Of note, postoperative intussusception in adults may also be caused by tube feeding which, to our knowledge, has rarely been reported before and only case reports.^[11,12]^ In the current study, we summarize the clinical data on a group of patients with tube feeding associated postoperative intussusceptions. The possible etiology and preventive measures will also be discussed.

## 2 Method

This is a single center retrospective case series study. The patients who received gastrointestinal anastomosis in the First Affiliated Hospital of Xi’an Jiaotong University during the period between May 2013 and January 2018, were retrospectively reviewed. Patients who received feeding tube and suffered from small bowel intussusception within 90 days after the surgery were included. The GJ tube (10F, liquid-capsule jejunum tube) we placed had a straight end with liquid capsule, and enteral nutrition liquid went through the side opening at the end of the tube. Normally, enteral nutrition with isocaloric and isonitrogenous enteral feed started on the first day post-operation at 25 ml/hour, with the rate increased if tolerated by 25 ml/hour every 24 hours. The GJ tube would be removed until oral food intake reached 60% of nutritional requirements.

Preoperative variables including standard demographic and pathological characteristics were collected. Details of the intussusception and its relationship with the GJ tube, such as feeding tube position, the interval from surgery to intussusception, symptoms, position, and direction of the intussusceptions were carefully reviewed. The etiological factors and clinical manifestations of tube feeding associated postoperative intussusceptions were carefully analyzed. After the surgery for intussusception, all patients were regularly followed-up and prospectively monitored for recurrence of intussusception every 6 months within the first 2 years, after which follow-up visit took place annually. All patients had signed the consent and this study was approved by the institution ethics committee of the First Affiliated Hospital of Xi’an Jiaotong University (No.XJTU1AF2019LSK-2019–062).

### 2.1 Statistical analysis

Continuous variables are expressed as medians with ranges or means with standard deviation (SD). Statistical analyses were performed using SPSS version 21.0 (IBM SPSS Inc., Chicago, IL, USA).

## 3 Results

Ultimately, among 1840 patients who received gastrointestinal anastomosis surgery, 7 patients (0.38%) had tube feeding associated intussusception and were enrolled into the study. Clinical and pathological characteristics of patients were summarized in Table 1. Most of the patients were male and the median age of the cohort was 58 years old (range 38–65). None of the patients was overweight before the first surgery.

**Table 1:**
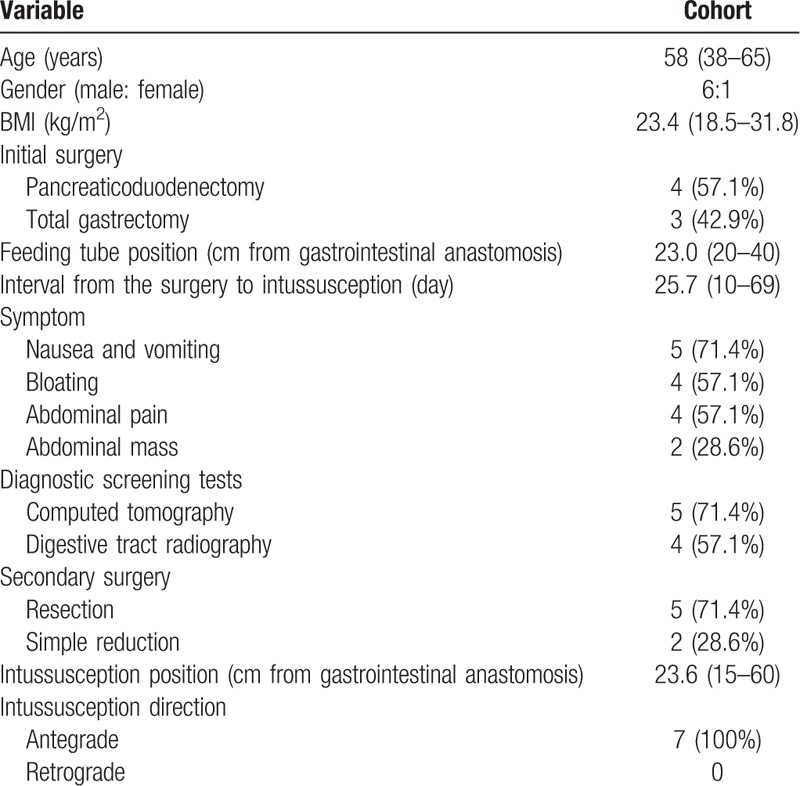
Clinical and pathological characteristics of 7 patients.

Some of the patients received open pancreaticoduodenectomy with Billroth II gastrointestinal reconstruction. The initial pancreatoduodenectomy was done without preservation of the pylorus (Kausch–Whipple) and then followed by Braun anastomosis. Those who received total gastrectomy had Roux-en-Y gastrointestinal reconstruction with no Braun anastomosis. GJ tube was placed during the reconstruction by inserting the liquid capsule into the efferent limb. The end of the feeding tube was 23.0 cm (range 20–40) from the gastrointestinal or Braun anastomosis.

Intussusceptions occurred from 10 to 69 days (median 25.7 days) postoperatively in an acute form. GJ tubes were not removed in all the patients because of delayed gastric emptying or recovery of gastrointestinal function after the operation.

**Figure 1:**
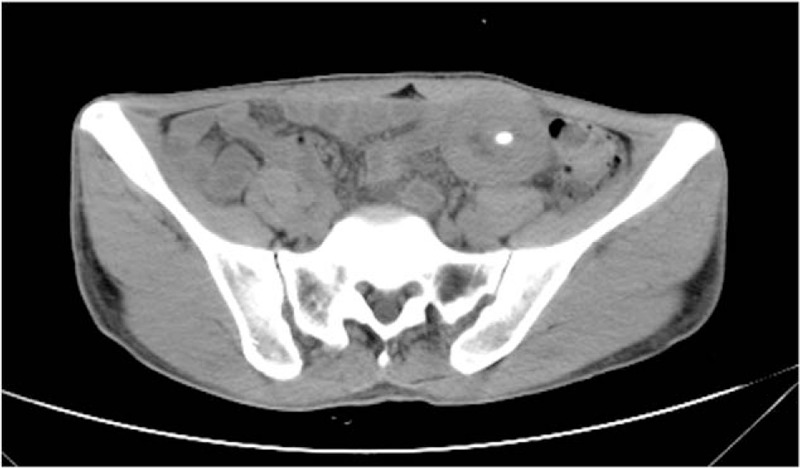
Transverse computed tomographic image showing the typical findings of postoperative gastrointestinal intussusception with feeding tube in the enteric cavity (high density shade).

Nausea and vomiting (5/7) were the major presenting symptoms for intussusceptions followed by bloating (4/7) and abdominal pain (4/7). Two patients presented with abdominal mass. None of the patient had fever, hematemesis, or melena. Intussusceptions were diagnosed by computed tomography in 5 patients and by fluoroscopy with contrast through the gastrojejunostomy in 4 patients. Signs suggesting upper gastrointestinal tract obstruction were present in 6 of the patients. The typical “target” sign which is highly suggestive of intussusception in CT scan could be found in 4 patients (Fig. 1). Radiological tests were normal in only 1 patient, in whom the diagnosis was made during surgical exploration.

None of the patient had spontaneous resolution of the intussusception. Gastroscope was attempted on 3 patients, whereas the intussusception did not reduce. Finally, all patients underwent surgery. Liquid capsule was found to remain filled in 2 patients. Antegrade efferent limb intussusceptions (type II jejunogastric intussusception) were found in all patients. Intussusception occurred at efferent loop and was 23.6 cm (range 15–60) from the gastrointestinal or Braun anastomosis. Ischemic segment of small bowel was found in 4 patients, but none with perforation. Five patients underwent resection including those with ischemia. Two received a simple reduction operation of the invaginated segment. The median operating time was 160 minutes (range 90–250). Postoperative courses were uneventful and none of the patients died.

Median follow-up time was 14 (range 7–64) months. None of the patients had recurrence of intussusception throughout the follow-up period.

## 4 Discussion

Postoperative gastrointestinal intussusception is a relatively rare but serious clinical entity, most commonly reported after gastric resection and gastrojejunostomy. It was first described by Bozzi in a patient with gastrojejunostomy in 1914.^[13]^ The first case of jejunojejunal intussusception after pancreatoduodenectomy was described by Sedgwick in 1970.^[14]^ The reported incidence of postoperative gastrointestinal intussusception is very low. Only 1 study reported a series of 23 patients in 2008.^[15]^ Despite its rarity, postoperative intussusception still needs to be considered in the differential diagnosis of bowel occlusion after gastrojejunostomy. In this paper, we report a group of patients with tube feeding associated postoperative intussusception with a morbidity of 0.38% which is similar to previous reports on other types of intussusception. However, the etiological factor and clinical manifestations of these patients are different, in some aspects, from the existing medical literature and reports.

### 4.1 Etiology

Adult intussusception is usually associated with the presence of a small tumor or polyp. However such lesions are hardly seen in patients with intussusception after gastrointestinal surgery. The mechanism of postoperative gastrointestinal intussusception is still unclear and both functional and mechanical causes could contribute. The hypothesis which was first put forward by Hocking et al suggests that intussusception after gastrointestinal surgery could be related to small bowel motility disturbances.^[16]^ These functional causes lead to dysmotility disorders including intestinal tract antiperistalsis, gastroparesis, and Roux stasis syndrome.^[4,17]^ It is thought to occur because of ectopic pacemakers in the bowel.^[4]^ Mechanical causes are usually associated with the surgery and postoperative conditions. One theory suggests that the weight loss after gastrointestinal surgery could facilitate the development of intussusception because of less resistance associated with the decreased thickness of the mesentery of the intussuscepted segment.^[3]^ Adhesion was also considered to be a contributing factor in the development of intussusception. Other mechanical causes include the diameter of gastrointestinal anastomosis, jejunal stenosis, long afferent limb, rise of abnormal abdominal pressure, functional disorder of ganglion cells, or neuron transmission after expanded bowel and long gastrostomy tubes.^[4,18]^

**Figure 2:**
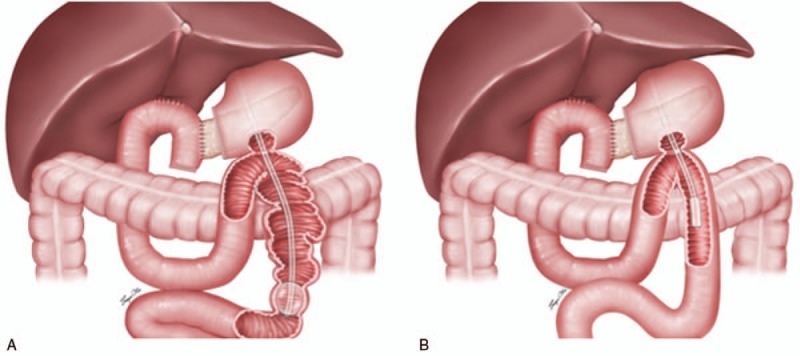
(A) Three possible explanations how GJ tube may cause postoperative gastrointestinal intussusceptions: GJ tube inserted into the efferent limb too deep; Length of the tube is shorter than natural length of the jejunum; Inflated balloon, or distal pigtail. (B) The way we suggest placing GJ tube which may prevent tube feeding related postoperative gastrointestinal intussusceptions.

Although the mechanism is complicated, patients in this study had the “lead point” which is highly associated with the setting of postoperative gastrointestinal intussusception, the GJ tube. Several possible explanations could be correlated with the findings in this case series (Fig. 2). Firstly, when GJ tube was inserted into the efferent limb too far away from the anastomosis, if there were other triggers that caused intestinal motility dysfunction, intussusception may have occurred at this segment of intestine based on the tube core effect similar to those caused by intestinal ascaris. Secondly, when the length of the GJ tube which is inserted into the efferent limb is shorter than natural length of the jejunum itself, the intestine may shrink over the GJ tube and more easily trigger intussusceptions. Thirdly, unsuitable enteral nutrient through the tube, mainly because of the low temperature or fast infusion, could cause intestinal peristalsis disorder and even cramps. Fourthly, some authors suggest that there may be restriction of peristalsis due to adhesion of mesentery or bowel in front of or behind the tip of the tube, and therefore repositioning of the inflated liquid capsule or distal pigtail could also cause intussusceptions.^[18]^ Finally, although mechanical causes such as weight loss and adhesion may not appear in the early postoperative period, most of the patients in our cohort suffered from intestinal motility disturbances and that is also why GJ tubes were not removed.

### 4.2 Clinical manifestations

According to the previous reports, intussusception can occur at any time after gastrointestinal surgery, varying from 6 days to 18 years.^[4]^ The average interval from surgery to intussusception is about 6 years.^[15,19]^ However, in the current study, median time from surgery to the onset of intussusception is 25.7 days. This means that tube feeding associated postoperative intussusception is more likely to occur as an early postoperative complication and mostly in the acute form. The risk of the tube feeding associated intussusception after Roux-en-Y gastrointestinal reconstruction in this study is comparable with the risk of 1 with Billroth II anastomosis. This is in contrast to Joshi study in which he found a higher morbidity of intussusception after Roux-en-Y gastrointestinal reconstruction.^[20]^

Postoperative gastrointestinal intussusception can be anatomically classified into 5 types according to the relationship with the jejunal loop, including afferent limb intussusception (type I), efferent limb intussusception (type IIa), efferent-efferent intussusception (type IIb), a combination of afferent and efferent limb intussusception (type III), intussusception through a Braun anastomosis (type IV).^[4]^ Similar to the existing scholarly literature, all our cases were efferent limb (Type IIb) intussusceptions.^[21,22]^ It is consist with the reports before that most cases with the efferent limb involved were acute intussusceptions.^[23]^ The difference is that in the previous literature, the most common type of intussusception after gastric surgery is retrograde intussusception,^[3,24]^ whereas all the tube feeding associated postoperative intussusception in our study are antegrade intussusceptions.

The symptoms of postoperative gastrointestinal intussusception are not specific and clinical presentations can be varied. It can still be classified into 2 forms according to the clinical manifestations: acute form and chronic form. As we described, all patients in this study suffered from an acute form gastrointestinal intussusception. Symptoms similar to upper intestinal obstruction were present. More than two thirds (71.4%) of the patients presented with nausea/vomiting. Abdominal pain was constant and mostly distension pain. Palpable mass, which is encountered in 2 out of 7 patients in the current study, should also be suggestive of the tube feeding associated postoperative intussusception.

It has been suggested that CT scan is currently the most useful radiological test for the diagnosis of intussusceptions with the typical target sign in about 80% of cases.^[25]^ Contrast radiography study and transabdominal ultrasonography are also relatively safe and noninvasive methods.^[26]^ For tube feeding associated postoperative intussusceptions, the radiological sign of the GJ tube could indicate the location of intussusceptions. In the current study, we were able to see the typical sign of CT scan along with the tube near or in the concentric annulus intestinal loop as another definitive factor. Similarly, the tube could also be seen passing through the center of the intestine lumen in the contrast radiography study. In ultrasound image, it was reported that the GJ tube could be represented by an echogenic area casting a shadow across the intussusception.^[10]^

### 4.3 Treatment

The treatment of postoperative gastrointestinal intussusception remains controversial.^[3]^ Although intussusception may resolve spontaneously, it is rarely seen in those after gastrointestinal surgery, especially the ones with the acute form. Treatment strategy should be decided once the diagnosis is made. It is reported that conservative management is discouraged and usually useless.^[27]^ Surgery should be indicated for patients with tube feeding associated postoperative intussusception because of the acute onset. The operation can be performed by laparoscopy or by laparotomy according to the clinical presentations and the experience of the surgeon.^[28]^ Simple reduction has been successfully reported by several studies, as in 3 of our patients; however recurrences should be born in mind.^[29–31]^ Some authors reported that gastrojejunal intussusception could be successfully reduced endoscopically in selected cases.^[32]^ However, in this study, we failed to reduce the intussusception by the non-surgical gastroscope strategy. According to studies, the nonviable segment of the jejunum including the gastrointestinal anastomosis should be removed in surgery and reconstruction is needed.^[3]^ For the tube feeding associated postoperative intussusceptions in our study, none of them involved gastrointestinal anastomosis. This may be due to the fact that the position of the GJ tube is usually placed more than 20 cm away from the anastomosis and leads to an efferent limb intussusception some distance away from the anastomosis. None of our patients has had recurrence when this current report is written, but longer-term follow-up would provide more information.

### 4.4 Prevention

Due to the unclear pathophysiological mechanisms of postoperative gastrointestinal intussusceptions, no effective preventative measure has been reported so far. Similarly in our study, Braun anastomosis was found to be ineffective as a preventative measure.^[4]^ Resection, rather than reduction, was recommended to prevent the recurrence of intussusception if the proximal jejunum had become dilated due to the restriction of bowel movement, and if there was adhesion of the mesentery or the bowel around the site of the first intussusception.^[12]^

For tube feeding associate postoperative intussusceptions, the following points should be considered. Position of the end of the GJ tube should be placed no more than 15 cm from the anastomosis. The length of the GJ tube which is inserted into the intestine should be equal to the natural length of the intestine, so that the intestine would not shrink and over fold around the GJ tube. The temperature of the enteral nutrient solution should be as close as possible to the body temperature, and the infusion rate should be slow and constant. Liquid in the capsule of the GJ tube must be removed after the tube is placed in position.

The current study had several limitations. The retrospective study design and the modest number of cases led to inherent selection bias. Secondly, the follow-up period of our research is relatively short which limit data on patients’ survival and longer-term complications.

## 5 Conclusion

Intussusceptions should be considered in patients with intestinal obstruction after gastrointestinal anastomosis surgery despite the low incidence. In contrast to other intussusceptions, the tube feeding associated postoperative intussusceptions have distinct etiological factors and clinical manifestations. It is more likely to occur in the early period after the surgery with an acute form. Surgical correction is recommended for most patients. Some measures have been proposed to prevent such complications after gastrointestinal surgery; however more studies and information are still needed.

## Author contributions

**Data curation:** Guozhi Yin.

**Formal analysis:** Dong Zhang.

**Project administration:** Jigang Bai.

**Supervision:** Zhimin Geng, Jigang Bai.

**Writing – original draft:** Liangshuo Hu, Guozhi Yin, Zhimin Geng.

**Writing – review & editing:** Liangshuo Hu, Jigang Bai.
